# Resilience to the Health Risks of Extreme Weather Events in a Changing Climate in the United States

**DOI:** 10.3390/ijerph8124582

**Published:** 2011-12-08

**Authors:** Kristie L. Ebi

**Affiliations:** Department of Medicine, Stanford University, 260 Panama Street, Stanford, CA 94305, USA; Email: krisebi@stanford.edu; Tel.: +1-650-521-3310

**Keywords:** adaptation, climate change, extreme weather events, health impacts, resilience

## Abstract

Current public health strategies, policies, and measures are being modified to enhance current health protection to climate-sensitive health outcomes. These modifications are critical to decrease vulnerability to climate variability, but do not necessarily increase resilience to future (and different) weather patterns. Communities resilient to the health risks of climate change anticipate risks; reduce vulnerability to those risks; prepare for and respond quickly and effectively to threats; and recover faster, with increased capacity to prepare for and respond to the next threat. Increasing resilience includes top-down (e.g., strengthening and maintaining disaster risk management programs) and bottom-up (e.g., increasing social capital) measures, and focuses not only on the risks presented by climate change but also on the underlying socioeconomic, geographic, and other vulnerabilities that affect the extent and magnitude of impacts. Three examples are discussed of public health programs designed for other purposes that provide opportunities for increasing the capacity of communities to avoid, prepare for, and effectively respond to the health risks of extreme weather and climate events. Incorporating elements of adaptive management into public health practice, including a strong and explicit focus on iteratively managing risks, will increase effective management of climate change risks.

## 1. Introduction

Public health has an impressive history of identifying and reducing health threats, as evidenced by the dramatic increase in life expectancy in the past 100 years. With more than 150 years of experience in identifying and responding to threats to health, public health should be well placed to address the health risks of climate change, including those posed by extreme weather and climate events (e.g., heatwaves, droughts, floods, windstorms). Initial policies and measures to adapt to the health impacts of climate change emphasized enhancing current health protection to climate sensitive health outcomes, such as implementing heatwave early warning systems [1]. Although such actions are vital, they will not necessarily reduce risks in a future climate; changing baselines mean that current efforts could be neutral or, in a worst case, maladaptive under new weather patterns. Further modifications are needed to increase the resilience of public health and health care policies and measures in a changing climate, where resilience is defined as the ability to timely and effectively anticipate, prepare for, respond to, and recover from climate change and other risks; definitions of resilience vary across disciplines, with all incorporating elements of effectively responding to, limiting impacts from, and recovering after a perturbation. The key difference from current public health practice is in explicitly acknowledging climate change in the design, implementation, monitoring, and evaluation of strategies, policies, and measures. Actions to reduce vulnerability to extreme weather events may not promote adaptation and resilience if they do not specifically consider the risks of climate change and how those risks could change over time.

Responsibility for the prevention of climate-sensitive health risks rests with individuals, community and state governments, national agencies, and others, with the roles and responsibilities varying by health outcome [2,3]. Actions to increase resilience across all actors include top-down (such as strengthening and maintaining disaster risk management programs) and bottom-up (such as through programs to increase social capital) measures, and need to focus not only on the risks presented by climate change but also on the underlying vulnerabilities that determine the extent and magnitude of impacts today and in the future.

Public health in the U.S. has historically focused on top-down measures aimed at reducing individual exposures and behaviors that increase disease risk. This approach is a consequence of public health tending to view the world through a medical model, with individuals and communities more as units for intervention than as partners in action. This framing of health results in programs that focus on individual-level factors that increase or decrease risk of adverse health outcomes, where risks typically are either addressed by changing individual risk factors (e.g., tobacco smoking, high cholesterol, and high blood pressure) or by top-down interventions (e.g., regulations to control emissions of air toxics). However, a growing body of research has clearly shown that community and social factors are significant predictors for a range of health outcomes [4]. The result has been increasing awareness that communities need to be active partners in identifying and addressing a wide range of health risks if public heath programs are to be effective and sustainable [5]. 

A challenge to increasing public health resilience to the health risks of climate change is that no single organization or institution has comprehensive authority and responsibility for creating, maintaining, improving, and evaluating the Nation’s public health infrastructure. Public health in the U.S. is promoted and delivered by a wide array of organizations and institutions, with highly variable resources, staffing, and performance capacity [6]. This is a consequence of a core value of the U.S. federal system: state and local autonomy and/or control, particularly in matters affecting personal risks and private interests. Each state is unique in its laws, regulations, governance, and budgets for public health, resulting in highly uneven abilities to deliver public health services.

There are significant opportunities for public health to increase the resilience of communities to the health risks of climate change. Mandates relevant to managing the risks of extreme weather and climate events are used as examples of opportunities for mainstreaming adaptation and increasing resilience, including (1) the all-hazards approach to health risks; (2) “healthy cities” programs; and (3) regionalization of public health services. Possible indicators for measuring public health preparedness and community resilience to extreme events also are discussed. 

Keim [7] identified the wide range of possible public health impacts of extreme events expected to worsen with climate change (Table 1). The impacts experienced in a particular location will vary, depending on the effectiveness of the public health and health care systems, and on the vulnerabilities specific to that location. 

**Table 1:** Relative public health impacts of selected extreme weather events. Source: Modified from [7].

Public Health Impact	Storms	Floods	Heat	Drought	Wildfire
Number of deaths	Few	Few, but can be many in flash floods	Moderate to high	Few	Few to moderate
Number of severe injuries	Few	Few	Moderate to many cases of heat stroke	Unlikely	Few to moderate
Worsening of existing chronic illnesses	Widespread	Focal to widespread	Widespread	Widespread	Focal to widespread
Increased pests and vectors	Widespread	Widespread	Unlikely	Possible	Unlikely
Risk of an epidemic	Unlikely	Unlikely	Unlikely	Unlikely	Unlikely
Food scarcity	Uncommon	Uncommon	Unlikely	Common	Possible
Loss of clean water	Widespread	Focal to widespread	Unlikely	Widespread	Focal
Loss of sanitation	Widespread	Focal to widespread	Unlikely	Likely among displaced populations	Likely among displaced populations
Loss and/or damage of health care systems	Widespread	Focal to widespread	Unlikely	Unlikely	Focal
Loss of shelter	Widespread	Focal to widespread	Focal to widespread	Focal to widespread	Focal
Permanent migration	Unlikely	Unlikely	Unlikely	Likely	Unlikely

## 2. All-Hazards Approach to Health Risks

An all-hazards approach to health risks is increasingly a foundation for organizing public health efforts to prepare for emergencies. These efforts could be modified to take into consideration the health risks associated with extreme weather events, including that the magnitude, extent, and duration of many types of events are increasing with climate change [8]. To increase resilience, the all-hazards approach would need to specifically incorporate the risks associated with climate change, and would need to have a strong monitoring and evaluation orientation to ensure that current risk management policies and measures are regularly revisited to ensure that they continue to be effective as the frequency and patterns of extreme events change.

The all-hazards approach is based on two laws that recognized weaknesses in state and local public health systems. The Public Health Improvement Act of 2000 calls for a plan to assure the preparedness of every community in the nation. The 2006 update and reauthorization of the Public Health Security and Bioterrorism Act, now called the Pandemic and All-Hazards Preparedness Act (Public Law No. 109-417) was intended “to improve the Nation’s public health and medical preparedness and response capabilities for emergencies, whether deliberate, accidental, or natural.” Funding is made available annually to meet a variety of goals, including developing and sustaining essential state, local, and tribal public health security capabilities, maintaining vital public health and medical services to allow for optimal operations in the event of a public health emergency, and addressing the needs of at-risk individuals.

A further mandate is the Department of Homeland Security National Preparedness Guidelines that define what it means for the nation to be prepared for any hazard [9]. The guidelines include national planning scenarios designed to focus contingency planning at all levels of government and in partnership with the private sector; included is a scenario for a major hurricane and for food contamination. The scenarios form the basis for coordinated Federal planning, training, exercises, and grant investments needed to prepare for emergencies. There is a universal task list with more than 1,600 unique tasks to facilitate efforts to avoid, prepare for, respond to, and recover from major events. In addition, 37 specific capabilities are listed that communities, the private sector, and all levels of government should collectively possess to respond effectively to disasters. The National Preparedness System then describes the actions needed at all levels of government, the private sector, nongovernmental organizations, and individuals to work together to achieve the priorities and capabilities outlined in the Guidelines. The Department of Homeland Security followed issuance of these guidelines with Presidential Directive 21 that established a National Strategy for Public Health and Medical Preparedness [10]. This directive focused on threat awareness, prevention and protection, surveillance and detection, and response and recovery, which are all highly relevant to emergency management of extreme weather events.

In response to these laws and other mandates, the U.S. Centers for Disease Control and Prevention (CDC) has developed performance measures, targets, definitions, instructions, data collection, and submission methods to demonstrate the degree of state, local, and tribal all-hazards preparedness [11]. Much of the data necessary to measure and monitor preparedness can be obtained during commonly occurring events, such as infectious disease outbreaks. In addition, state, local, and tribal entities who receive funding for all-hazards preparedness are expected to conduct drills and exercises to ensure that information is available for each performance measure. For example, one activity developed to support planning and local jurisdictions is the Cities Readiness Initiative to prepare major U.S. cities and metropolitan areas to effectively respond to a large-scale bioterrorist attack by dispensing antibiotics to their entire identified population within 48 hours of a decision to do so [12]. According to CDC, developing and maintaining the capacity to achieve this goal has enhanced communication and collaboration across state and local boundaries, and has helped local and state planners identify capabilities, strengths, and shortcomings through preparedness planning and technical assistance reviews. Because not all communities are involved, the overall level of preparedness can vary significantly within and across regions.

Based on performance measures and experiences, states have undertaken activities to improve their preparedness. For example, the Mississippi State Department of Health did not have the medical surge capacity to care for the thousands of individuals with special medical needs who were displaced during Hurricane Katrina [11]. All-hazards funding has since been used to equip buildings on selected campuses to act as special medical needs shelters for use in the event of a storm, a pandemic outbreak, or other natural or man-made disaster. In addition, alterations to electrical power systems will enable climate control and life support systems in the event of a power loss, and facilities are being retrofitted for use by physically challenged individuals. As evidence of progress, the Department of Health recently used the Mississippi Health Alert Network to notify the state’s healthcare system of a serious outbreak of pertussis [11]. One person notified every participating physician, hospital, and other medical care provider in about six hours, with a verified delivery rate of 90%. Previously, such contact took a minimum of 12–14 hours with a 50% success rate. This capacity will be highly valuable in the event of another storm surge or other climate-related event.

CDC created a Local Public Health Preparedness and Response Capacity Inventory for rapid assessment of a public health agency’s ability to respond to public health treats and emergencies [13]. The focus areas are preparedness, planning and readiness, surveillance, laboratory capacity, communication, and training. To assist local health departments in meeting preparedness mandates, the National Association of County and City Health Officials (NACCHO) developed a competency-based training and recognition program, Project Public Health Ready (PPHR) with three project goals; one is all-hazards preparedness planning [14]. The PPHR criteria are continuously updated to include the most recent federal initiatives. In addition, NACCHO provides a toolkit of best practices, self-assessments for local health departments, guidance on regional approaches to preparedness, and evaluations.

In addition to these activities, there are a growing number of methods and tools developed by a wide range of organizations to facilitate preparedness, including the Association of State and Territorial Health Officials (ASTHO), Council of State and Territorial Epidemiologists, National Association of Local Boards of Health, Association of Public Health Laboratories, Agency for Healthcare Research and Quality, and many others. One example is an online map-based planning tool developed by the Western New York Public Health Alliance Practice Center with its partners, the National Opinion Research Center at the University of Chicago and the Pennsylvania State University Center for Environmental Information (www.cei.psu.edu/evac). The tool assumes that when a disaster strikes an urban area, a significant number of residents will self-evacuate to surrounding rural communities. The tool allows users to select a city of interest and model how communities within a 150-mile radius might be affected by the spontaneous evacuation of urban residents following a dirty bomb explosion, chemical incident, or influenza pandemic. Maps show numbers of evacuees received by each surrounding county and their resulting population changes. Information on the number of hospital beds, hotel rooms, and other resources can be used to determine the resources a region may need to respond to evacuee and resident needs. Such tools could be modified to increase their relevance for other communities and in other contexts. 

These and other initiatives are intended to improve public health preparedness to the types of impacts and disasters that are projected to increase with climate change, although they have yet to explicitly take climate change into account. For example, in 2001, some state public health departments did not have enough epidemiologists to investigate suspected anthrax cases, and had not fully anticipated the extent of coordination needed among first responders [11]. By 2007, in every state, cooperative agreements supported additional staff, public health departments had established relationships and conducted exercises with emergency management and other key players, and emergency operations centers were in place at CDC and almost all state public health departments to coordinate response activities. As noted earlier, these public health programs can be modified to specifically include the health risks of climate change, with strong monitoring and evaluation to ensure effectiveness as situations change.

However, an independent analysis of the Nation’s preparedness for public health emergencies found that although significant progress has been made, there are important areas where continued and concerted actions are needed [15]. On a range of measures, federal and state polices still fall short of stated goals. Variation in preparedness among the states results in different levels of protection from potential emergencies. The report concluded there is a need to increase accountability, strengthen leadership; enhance surge capacity and the public health workforce; modernize technology and equipment; and improve community engagement. These are all determinants of adaptive capacity. For example, an evaluation of 24/7 critical case reporting that compared self-reports with operational data found that 18 of 19 local health department in a convenience sample indicated that they had a process in place to receive and respond to calls on a 24/7 basis, but operational assessments found that only two consistently met the prescribed standard [16]. 

A key problem is that assessments tend to focus on public health structures, but there is little evidence that links specific preparedness structures with the ability to execute effective responses to protect population health [17]. Focusing only on public health structures misses the additional factors that affect resilience, such as the social capital needed for effective community response to disasters and threats; programs need a broader basis that incorporates all relevant agencies and departments. It also misses changes that are needed to urban social-ecological systems, through urban planning and design standards for the built environment, to lower risks to individuals and communities when affected by an extreme event. 

Modifying the goals and approaches of all-hazards programs to incorporate the changing risks associated with alterations in the frequency, intensity, and duration of extreme events, would increase preparedness today and under future climates. 

## 3. Regionalization of Health Services

Since 2001, there has been a national emphasis on increasing emergency preparedness for possible terrorist attacks by regionalizing local services, where regionalization is the addition of a regional structure to supplement local government agencies. Although the US relies on state and local health departments to provide essential public health services, few counties, cities, or towns are capable of responding to health emergencies on their own. For example, a national survey conducted in 2000–2001 found that only 25% of local public health jurisdictions felt they were able to deliver at least 60% of the essential public health services in the event of a terrorist attack [18]. In response, there has been increasing regionalization of public health services [19]. 

Rationales for regionalization include reducing socioeconomic and fiscal disparities between metropolitan and outlying areas [20]; creating economies of scale; and sharing human and financial resources regionally to improve efficiency and consistency of delivery of public health services, as well as reduce duplication of efforts, across local, regional, and state levels [21]. The potential benefits include improving both public health preparedness and response, as well as creating social capital. NACCHO, through its Project Public Health Ready program, identified four approaches to facilitating effective regionalization [14]: networking through the interactive sharing of preparedness information and plans between individuals and organizations; coordinating local health departments for planning events, such as training or exercises; standardizing across local health departments through mutual adoption of emergency preparedness functions; and centralizing resources for planning and response under a single entity, such as a single web portal, an emergency notification systems, or regional epidemiologic support.

Lessons learned from case studies of public health responses to outbreaks of West Nile virus, SARS, monkeypox, and hepatitis A in the U.S. illustrate the value of regionalization to increase preparedness [22]. One lesson was that the required public health response is not necessarily proportional to the number of people actually exposed, infected or ill, or the number of deaths. This is because public health efforts to identify additional cases (particularly of cases of infectious diseases) through active surveillance are likely to result in the identification of many potential cases, including individuals who do not have the disease but are worried that they do. Extensive population-based prevention efforts, such as education campaigns, may be necessary to limit disease transmission and reduce the health consequences. These demands stress the capacity of public health systems even when the actual number of cases is small, such as a disease outbreak following a flooding event. Therefore, increased sharing of human and financial resources can increase the capacity of local public health agencies to cope when challenged with an outbreak or emergency.

Worldwide and national attention on increasing preparedness for pandemic influenza has increased interest in building regional surge capacity [21] including centralizing staff, space, and supplies. The lack of surge capacity was one of the current constraints to adaptation noted in the climate change adaptation assessment conducted by the Maryland Commission on Climate Change [23]. 

An example is Massachusetts, which regionalized its 351 autonomous cities and towns, each with its own local health department or board of health (as required by state law), into public health emergency preparedness regions and sub-regions to more effectively distribute CDC funding for state preparedness [21]. One action taken was to implement regional training programs, which advanced an iterative cycle of developing plans, training personnel, testing preparedness, and refining plans to clarify specific roles and responsibilities [24]. 

Regionalization is an opportunity to facilitate sharing of knowledge and resources to prepare for and respond to climate sensitive health threats. A challenge is that public health organizations and institutions tend to spend much of their time dealing with health threats as they occur. Chronic underfunding means that recognized gaps in preparedness often are not addressed until there are adverse health outcomes, after which there will be funding for a few years before funds are diverted to the next critical need [25].

Other challenges to increasing regionalization include that little is known about the best ways to define a region and what are the best practices [21]. Barriers to regionalization include fear of the loss of local autonomy and identity; lack of formal legal agreements that address liability and mutual aid; and resources to participate in regional activities. Public health agencies typically do not have command and control authority over important resources, such as hospitals, health care providers, and other government agencies, that are needed for an optimal public health response [22]. At the same time, there needs to be effective communication and coordination during a public health emergency. Jurisdictional arrangements can be complicated across state, regional, county, and city entities, each of which operates under different political leadership structures and local governmental and community organizations. Clearly, regionalizing public health services needs to address authority issues and complex jurisdictional arrangements. But addressing these challenges can increase resilience of communities and states to a range of risks, including extreme weather events associated with climate change.

## 4. Healthy Cities Programs

Urban health assesses the impact of urban living on health through the physical environment, the social environment, and access to heath and social services [26]. Programs to promote urban health have generally focused on specific problem areas or populations. One widespread program is Healthy Cities, which is a community problem-solving process for health promotion. WHO defines a Healthy City [27] as “*one that is continually creating and improving those physical and social environments and expanding those community resources which enable people to mutually support each other in performing all the functions of life and developing their maximum potential.*”

The Healthy City (now called Healthy Communities) process involves encouraging community participation, assessing community needs, establishing priorities and strategic plans, soliciting political support, taking local action, and evaluating progress [28]. The initial goals were to address primary health care and health promotion. It challenged communities to develop projects to reduce inequalities in health status and access to services, and to develop healthy public policies at the local level through a multi-sectoral approach and increased community participation in health decision-making. The basis of the European WHO Healthy Cities is that inequalities in social conditions (and therefore health) are unjustified and that their reduction should be an overriding public health objective [27]. Decreasing population vulnerability and building social capital provide a strong basis for increasing resilience to the health risks of extreme weather events.

The Healthy Cities programs typically view health broadly, and develop projects to collaboratively address the root causes of problems [27]. Projects are initiated independently, based on a range of philosophical orientations and with different sponsoring organizations and funding. CDC, NACCHO, and others provide information and tools for Healthy Cities projects. Common areas of action include the environment, community safety, immunizations, tobacco, and youth. Some of the characteristics that make a city a Healthy Cities include effective leadership; a multi-sectoral committee or steering group that directs the project; a strong economy; community participation; information used in citywide planning; needed technical support obtained; program viewed as a credible resource for health in the community; effective networking; and smaller city size or neighborhoods in large cities [28]. These factors also facilitate effective adaptation. Although some of these characteristics are beyond the control of the local community, many can be advanced through community partnerships. Given the variety of ways in which these characteristics can be measured, and the large number of interactions that could increase or decrease resilience, there is no consensus on the best indicators for designating a city as a “Health City”. 

Glouberman *et al*. [29] propose a framework that combines the urban health and Healthy Cities approaches into a Health in Cities process that recognizes the particular vulnerabilities and problems faced by specific populations within an urban environment and the effects of the urban environment on all city residents (*i.e.*, that the health of a city is a multi-directional interaction between the residents and the environment). They advocate approaching health promotion in cities from the perspective of complex adaptive systems; that is, the entire system can be viewed as a network of relationships and interactions. This is a key component of adaptive management. Successful strategies for change in cities will not succeed without understanding the local context. Similar conclusions have been reached in assessing the adaptive capacity of communities to address the risks of climate change.

It is possible to build on the Healthy Cities or Health in Cities approaches to address the health risks of climate change [30]. Local actions aimed to reduce vulnerability and increase adaptive capacity can be designed to also reduce greenhouse gas emissions through alterations in transport policies and other actions [3]. Ebi and Semenza [5] outline a framework for facilitating community-based adaptation to the health impacts of climate change, with each step designed to enhance social capital and build resilience. 

## 5. Possible Indicators of Community Resilience to Extreme Events

Assessing the ability of communities to prepare for and effectively respond to the health impacts of extreme events requires identification of a limited set of metrics for regular measurement and monitoring, either as mandated by the relevant agencies or by extending their mandate. General indicators of community resilience to extreme weather events associated with climate change can be categorized into (1) indicators of public health preparedness; (2) indicators of health-outcome specific morbidity and mortality; and (3) indicators of socioeconomic, political, technical, infrastructure, and other vulnerabilities. 

English *et al*. [31] identified climate change and health indicators for the United States that were chosen to describe elements of environmental sources, hazards, exposures, health effects, and intervention and prevention activities. Some indicators are measures of environmental variables that can directly or indirectly affect human health, such as maximum and minimum temperature extremes, while others can be used to project future health impacts based on changes in exposure, assuming exposure-response relationships remain constant. Indicators were categorized into four areas: environmental, morbidity and mortality, vulnerability, and policy responses related to adaptation and greenhouse gas mitigation. These indicators cover measures that, if monitored, would track the magnitude and extent of health impacts.

Developing indicators for evidence-based measures of preparedness for a range of disasters is limited by the rarity of large-scale public health emergencies, and because other agencies typically have primary responsibility for emergencies. Using the ten essential public health services, CDC established a National Public Health Performance Standards Program to improve the quality of public health practice and the performance of public health systems [32]. The program is a collaborative effort of seven national partners that describes the public health activities that should be undertaken at state and local levels. It provides web-based tools and resources for state and local public health systems to self-assess and improve their performance. The local public health system assessment focuses on all entities that contribute to public health services within a community, while the local public health governance assessment focuses on the local governing bodies accountable for public health (*i.e.*, boards of health, councils, or county commissioners). For state public health system assessments, there should be planning and implementation; state-local relationships; performance management and quality improvement; and public health capacity and resources for each essential service. For local public health governance, there should be oversight of the activities undertaken by local public health systems. The standards developed do not explicitly address the health risks of climate change, but easily could with the development of indicators for monitoring the risks posed by climate change and the effectiveness of programs and activities to increase current and future community resilience. 

Indicators of key determinants of health outcomes need to include factors that influence the ways that social, environmental, behavioral, and health services determine population health [33]. Populations living in certain regions may have increased risks for specific climate-sensitive health outcomes due to their regions’ baseline climate, abundance of natural resources such as fertile soil and fresh water supplies, elevation, dependence on private wells for drinking water, and/or vulnerability to coastal surges or riverine flooding [34]. Socioeconomic factors interact with the physical threats of extreme weather events and other disasters in determining vulnerability and sensitivity; they may increase the likelihood of exposure to harmful agents, interact with biological factors that mediate risk, and/or lead to differences in the ability to adapt or respond to exposures or early phases of illness and injury [34]. 

In addition to indicators of socioeconomic, physical, and other vulnerabilities to the health risks of climate change, there is growing recognition that communities are complex systems that are inadequately described in the traditional medical paradigm where risk factors have unidirectional associations with adverse health outcomes [35]. The dynamics of communities have been shown to determine health in their own right, independent of an individual’s health and socioeconomic status. In addition, interventions that did not incorporate a broader understanding of the population in which they are being implemented (e.g., cultural context, social norms) have had limited success [36]. As in ecology, multidirectional relationships may more effectively describe the vulnerability and resilience of the population. Effectively addressing vulnerabilities to the health risks of climate change requires not only interventions to reduce specific health vulnerabilities, but also the societal, cultural, environmental, political, and economic context that increases vulnerability [37,38]. Engaging communities in the process of adaptation will not only enhance their resilience to climate stressors, but also may increase their ability to cope with a wide range of other societal issues [5], as evidenced by successes with the Healthy Cities programs.

## 6. Conclusions

Individuals and communities resilient to climate change anticipate risks; reduce vulnerability to those risks; prepare for and respond quickly and effectively to threats; and recover faster, with increased capacity to prepare for and respond to next threat. Public health institutions are structured to anticipate and prepare for risks, reduce identified vulnerabilities, and rapidly respond through a wide range of programs and activities, and have a long history of doing so effectively. However, preventing additional morbidity and mortality due to climate change will require modification of current and implementation of new programs and activities to increase resilience to climate change, taking into consideration the local context, including socioeconomic, geographic, the built environment, and other factors [2]. Programs designed for other purposes provide opportunities for increasing the capacity of public health organizations and institutions to avoid, prepare for, and effectively respond to the health risks of extreme weather and climate events.

Incorporating elements of adaptive management, a structured and iterative process of decision-making in the face of imperfect information, into public health practice will increase its effectiveness to address the health risks of climate change, through a strong and explicit focus on iteratively managing risks [39]. 

A critical challenge is to enhance the capacity of public health to increase its own and facilitate community resilience in less-than-robust environments [6]. Public health infrastructure has been seriously and systematically underfunded owing to its low priority among state and federal policy makers. A survey in the late 1990s, before passage of the Public Health Improvement Act and the Pandemic and All-Hazards Preparedness Act, found total per-capita spending on essential public health services of $37–$102 among local agencies and $86–$232 among state agencies [25]. There were subsequent improvements in public health funding, but funding is subject to changing priorities and is unevenly distributed across regions and issues of concern, leaving too many communities vulnerable to the extreme events associated with climate change. 

Despite this, considerable improvement in community resilience can be expected as key public health agencies and organizations, such as the Department of Health and Human Services, CDC, NACCHO, ASTHO, the Red Cross, and others, facilitate the inclusion of future climate change risks into current and planned strategies and programs, such as those highlighted. In addition to traditional approaches to improving the public health infrastructure and systems, the social-ecological determinants of health should be explicitly included when identifying opportunities for increasing resilience to the health risks of climate change.
